# Exploring the limits of localization: federated model stacking improves hospital-level prediction in a national research network

**DOI:** 10.1038/s41746-026-02634-1

**Published:** 2026-04-24

**Authors:** Jie Cao, Emily Balczewski, Rahul Ladhania, Paramveer Dhillon, Donald S. Likosky, Allison M. Janda, MPOG Collaborators, Nathan L. Pace, Robert E. Freundlich, Douglas A. Colquhoun, Bradley A. Fritz, Robert B. Schonberger, Sachin Kheterpal, Michael R. Mathis, Karandeep Singh

**Affiliations:** 1 Jacobs Center for Health Innovation, UC San Diego Health, San Diego, CA USA; 2 Department of Computational Medicine and Bioinformatics, University of Michigan Medical School, Ann Arbor, MI USA; 3 Department of Health Management and Policy, University of Michigan, Ann Arbor, MI USA; 4 School of Information, University of Michigan, Ann Arbor, MI USA; 5 Department of Cardiac Surgery, University of Michigan Medical School, Ann Arbor, MI USA; 6 Department of Anesthesiology, University of Michigan Medical School, Ann Arbor, MI USA; 7 Division of Biomedical Informatics, Department of Medicine, University of California, San Diego, CA USA; 8 Department of Anesthesiology, University of Utah, Salt Lake City, UT USA; 9 Department of Anesthesiology and Critical Care, Vanderbilt University Medical Center, Nashville, TN USA; 10 Department of Anesthesiology, Washington University in St. Louis School of Medicine, St. Louis, MO USA; 11 Department of Anesthesiology, Yale School of Medicine, New Haven, CT USA

**Keywords:** Health care, Medical research

## Abstract

Challenges with model generalizability and data privacy have led to a shift in health artificial intelligence (AI) models being trained locally within individual health systems rather than relying on multicenter data. Localization carries the promise of capturing local practice patterns and patient demographics, presumably resulting in better models. Our study empirically tests this hypothesis in a national research network by comparing locally trained models predicting acute kidney injury (AKI) after cardiac surgery with two multicenter modeling approaches, pooling and a novel federated model stacking method. Trained on 43,926 cases across 23 hospitals, the study finds that multicenter models outperform single-center approaches, with higher area under the receiver operating characteristic curves (AUCs) for all AKI severity levels in both temporal and external validation sets. Hospitals with smaller case volumes benefit the most from multicenter approaches, showing the greatest AUC increase over locally trained models.

## Introduction

Challenges with model generalizability across health systems have led to a recent shift in health artificial intelligence (AI) models being trained locally within individual health systems rather than relying on multicenter data. Localizing models to their intended environment carries the promise of capturing relationships between local practice patterns, patient demographics, and health outcomes^1,2^, presumably resulting in better AI models. The practice of localizing AI models has been facilitated by implementation of the local retraining process into the electronic health record^3^ and further burgeoned by growing privacy and security concerns around multicenter data sharing driven by a rise in ransomware attacks against health systems^4,5^. However, localization also has the potential to produce worse models when training data lack robust sample size or diversity^6,7^.

At the same time that the adoption of localization has been rising, two developments have made multicenter modeling more feasible and secure: federated learning and deidentified data aggregation networks. Federated learning refers to the practice of training models across health systems without directly sharing patient data^8^. Federated learning reduces the privacy risks associated with data sharing and has been shown to work in research contexts, including intensive care unit (ICU) outcomes^9,10^, COVID-19 outcomes^11–13^, breast density classification^14^, and rare cancer boundary detection^15^, but its adoption in clinical settings has been limited. While sharing and pooling patient data carries risks, a number of vendors have recently developed national data aggregation platforms that mitigate these through deidentification^16,17^.

Given the tradeoffs between localization (i.e., local retraining) and multicenter modeling, the right approach may depend on the context, such as whether a health system is large or small. Prior methods supporting the decision on whether to localize a model have relied on statistical tests, which require access to the underlying data from a health system and may not be feasible^18^ Rigorous empirical evidence supporting the right approach to localization is lacking.

Our study empirically tests the benefits of localization in a national research network comprising 31 academic and community hospitals by comparing locally trained models predicting acute kidney injury (AKI) after cardiac surgery with two multicenter modeling approaches, pooling and a novel federated model stacking method. We evaluate whether single-center local models outperform multicenter approaches, and if the optimal approach depends on the number of hospitals within a research network. Given the limited adoption of federated learning approaches due in part to their complexity, we use a novel two-stage federated model stacking method for federated learning rather than sequential approaches that require multiple rounds of retraining to achieve convergence.

## Results

### Cohort characteristics

We identified a total of 66,166 cardiac surgery cases across 31 US academic and community hospitals meeting inclusion criteria (Supplementary Fig. 1). Of these, 43,926 cases across 23 hospitals (*n* = 43,926) were included in the training set, 18,132 across 25 hospitals in the temporal validation set, and 4,108 cases across 4 hospitals in the external validation set (see Supplementary Table 1 for details). Our overall cohort had a mean age of 62.0 ± 13.5 years old and consisted of more males (68.4%) and non-Hispanic whites (79.6%) (Table 1). Surgical cases had a mean duration of 6.98 ± 2.22 h in the operating room and 2.33 ± 1.47 h of cardiopulmonary bypass (CPB). The temporal validation set had similar baseline characteristics as the training set, except for more non-smokers (29.1% vs 18.6%) and a higher burden of comorbidities (Supplementary Table 2). As compared to the training set, the external validation cases were older (mean age 64.4 ± 12.3 vs 61.9 ± 13.6), were less diverse in regard to sex, race, and ethnicity (males 72.4% vs 68.0%, non-Hispanic whites 84.9% vs 79.3%), had fewer comorbidities, and had shorter operating room duration (6.19 ± 2.02 h vs 6.95 ± 2.22 h).

**Table 1:** Summary patient and surgical characteristics

Characteristic	Overall (*N* = 66,166)	Training (*N* = 43,926)	Temporal validation (*N* = 18,132)	External validation (*N* = 4108)
Preoperative patient characteristics				
Age (years)	62.0 (13.5)	61.9 (13.6)	61.7 (13.3)	64.4 (12.3)
Sex				
Female	20,921 (31.6%)	14,062 (32.0%)	5726 (31.6%)	1133 (27.6%)
Male	45,245 (68.4%)	29,864 (68.0%)	12,406 (68.4%)	2975 (72.4%)
Race/Ethnicity				
White not of Hispanic origin	52,643 (79.6%)	34,822 (79.3%)	14,335 (79.1%)	3486 (84.9%)
Black not of Hispanic origin	4304 (6.5%)	2720 (6.2%)	1494 (8.2%)	90 (2.2%)
Asian or Pacific Islander	2068 (3.1%)	1215 (2.8%)	624 (3.4%)	229 (5.6%)
Bi or Multi Racial	569 (0.9%)	413 (0.9%)	156 (0.9%)	0 (0.0%)
American Indian or Alaska Native	180 (0.3%)	102 (0.2%)	66 (0.4%)	12 (0.3%)
Hispanic white	514 (0.8%)	267 (0.6%)	206 (1.1%)	41 (1.0%)
Hispanic black	38 (0.1%)	18 (0.0%)	19 (0.1%)	1 (0.0%)
Middle Eastern	38 (0.1%)	38 (0.1%)	0 (0.0%)	0 (0.0%)
Missing	5812 (8.8%)	4331 (9.9%)	1232 (6.8%)	249 (6.1%)
Height (cm)	171.9 (14.1)	172.5 (10.9)	170.2 (19.9)	172.4 (10.4)
Weight (kg)	87.0 (20.8)	86.7 (20.7)	87.7 (21.2)	87.2 (21.0)
Body Mass Index (kg/m^2^)	29.1 (6.2)	29.0 (6.2)	29.2 (6.3)	29.3 (6.3)
ASA physical status classification				
ASA Class 1	59 (0.1%)	54 (0.1%)	3 (0.0%)	2 (0.0%)
ASA Class 2	397 (0.6%)	267 (0.6%)	72 (0.4%)	58 (1.4%)
ASA Class 3	13,838 (20.9%)	8806 (20.0%)	3130 (17.3%)	1902 (46.3%)
ASA Class 4	50,982 (77.1%)	34,286 (78.1%)	14,614 (80.6%)	2082 (50.7%)
ASA Class 5	890 (1.3%)	513 (1.2%)	313 (1.7%)	64 (1.6%)
Preoperative laboratory values				
White Blood Cell Count (per mL)	7.6 (3.1)	7.5 (3.1)	7.7 (3.1)	7.9 (3.4)
Platelet Count, (K/mL)	218.6 (72.2)	216.9 (70.2)	221.9 (77.2)	223.0 (70.4)
Hemoglobin (g/dL)	13.3 (2.0)	13.3 (2.0)	13.2 (2.1)	13.3 (1.9)
Sodium (mEq/L)	138.8 (3.2)	139.1 (3.2)	138.3 (3.1)	138.5 (3.1)
Potassium (mEq/L)	4.2 (0.4)	4.2 (0.4)	4.2 (0.4)	4.1 (0.4)
Bicarbonate (mmol/L)	25.6 (3.1)	25.8 (3.1)	25.3 (3.3)	24.8 (2.8)
Glucose (g/dL)	115.8 (39.3)	115.1 (39.6)	116.5 (38.7)	119.0 (38.0)
Creatinine-related variables				
Preoperative baseline serum creatinine, g/dL	1.0 (0.3)	1.0 (0.3)	0.9 (0.3)	0.9 (0.3)
Preoperative most recent serum creatinine, g/dL	1.0 (0.5)	1.0 (0.6)	1.0 (0.3)	1.0 (0.3)
Preoperative serum creatinine ratio (most recent/baseline)	1.1 (0.5)	1.1 (0.6)	1.1 (0.2)	1.1 (0.2)
Preoperative serum creatinine difference (most recent - baseline)	0.1 (0.5)	0.1 (0.5)	0.1 (0.1)	0.1 (0.1)
First post-operative serum creatinine within 24 h	1.0 (0.3)	1.0 (0.3)	1.0 (0.3)	0.9 (0.3)
Preoperative AKI				
No preoperative AKI	62,831 (95.0%)	41,831 (95.2%)	17,081 (94.2%)	3919 (95.4%)
Preoperative AKI-1	3066 (4.6%)	1934 (4.4%)	967 (5.3%)	165 (4.0%)
Preoperative AKI-2	218 (0.3%)	136 (0.3%)	66 (0.4%)	16 (0.4%)
Preoperative AKI-3	51 (0.1%)	25 (0.1%)	18 (0.1%)	8 (0.2%)
Summary patient comorbidities (Elixhauser)				
Cardiac Arrhythmia	42,934 (64.9%)	27,245 (62.0%)	12,912 (71.2%)	2777 (67.6%)
Chronic pulmonary disease	15,116 (22.8%)	10,143 (23.1%)	4008 (22.1%)	965 (23.5%)
Coagulopathy	25,473 (38.5%)	15,989 (36.4%)	8898 (49.1%)	586 (14.3%)
Congestive Heart Failure	31,495 (47.6%)	19,952 (45.4%)	9910 (54.7%)	1633 (39.8%)
Diabetes	18,570 (28.1%)	11,661 (26.5%)	5321 (29.3%)	1588 (38.7%)
Fluid and electrolyte disorders	39,655 (59.9%)	25,740 (58.6%)	12,602 (69.5%)	1313 (32.0%)
Hypertension	24,791 (37.5%)	14,337 (32.6%)	8981 (49.5%)	1473 (35.9%)
Liver disease	4758 (7.2%)	2850 (6.5%)	1609 (8.9%)	299 (7.3%)
Peripheral vascular disorders	24,588 (37.2%)	15,781 (35.9%)	7557 (41.7%)	1250 (30.4%)
Pulmonary circulation disorders	12,034 (18.2%)	7682 (17.5%)	3632 (20.0%)	720 (17.5%)
Valvular disease	46,092 (69.7%)	31,145 (70.9%)	12,513 (69.0%)	2434 (59.3%)
Surgical characteristics - procedure type				
Valve only	21,670 (32.8%)	15,371 (35.0%)	5232 (28.9%)	1067 (26.0%)
Coronary artery bypass only	18,573 (28.1%)	11,350 (25.8%)	5356 (29.5%)	1867 (45.4%)
Aortic	9316 (14.1%)	6114 (13.9%)	2852 (15.7%)	350 (8.5%)
Valve + coronary artery bypass only	6455 (9.8%)	4433 (10.1%)	1492 (8.2%)	530 (12.9%)
Myectomy	2156 (3.3%)	1588 (3.6%)	541 (3.0%)	27 (0.7%)
Ventricular assist device	1680 (2.5%)	1161 (2.6%)	463 (2.6%)	56 (1.4%)
Heart transplant	1669 (2.5%)	953 (2.2%)	664 (3.7%)	52 (1.3%)
Pulmonary thromboendarterectomy	385 (0.6%)	242 (0.6%)	143 (0.8%)	0 (0.0%)
Other	4264 (6.4%)	2716 (6.2%)	1389 (7.7%)	159 (3.9%)
Additional surgical characteristics				
Anesthesia duration (min)	419 (133)	417 (133)	434 (134)	371 (121)
Cardiopulmonary bypass duration (min)	140 (88)	133 (85)	157 (99)	137 (65)
Emergent	4059 (6.1%)	2613 (5.9%)	1165 (6.4%)	281 (6.8%)
Institutional characteristics				
Academic Hospital	64,920 (98.1%)	43,198 (98.3%)	18,111 (99.9%)	3611 (87.9%)
Outcome characteristics				
CSA-AKI stage				
No CSA-AKI	49,264 (74.5%)	33,019 (75.2%)	12,901 (71.2%)	3344 (81.4%)
CSA-AKI-1	11,759 (17.8%)	7771 (17.7%)	3449 (19.0%)	539 (13.1%)
CSA-AKI-2	3457 (5.2%)	2161 (4.9%)	1142 (6.3%)	154 (3.7%)
CSA-AKI-3	1686 (2.5%)	975 (2.2%)	640 (3.5%)	71 (1.7%)

Statistics presented as mean (SD) for numeric variables; *N*(%) for categorical variables. *AIDS/HIV* acquired immunodeficiency syndrome/human immunodeficiency virus, *AKI* acute kidney injury, *ASA* American Society of Anesthesiologists, *CPB* cardiopulmonary bypass, *CSA-AKI* cardiac surgery-associated acute kidney injury, *ETT* endotracheal tube, *LMA* laryngeal mask airway.

^a^Non-mutually exclusive.

Among all cases in the overall cohort, 25.5% developed any AKI: 24.8% in the training set, 28.8% in temporal validation, and 18.6% in external validation. The median institution-level AKI was 29.7%, with an interquartile range of 23.6 to 34.0%. Prior to surgery, 5.0% of included cases had preoperative AKI, which was similar across the cohorts.

### Aggregate model performance

In the temporal validation set, the pooled models demonstrated the highest AUCs for all AKI severity levels (AKI 1+: 0.856; AKI 2+: 0.890; AKI 3+: 0.911), while single-center base models had a lowest AUCs (AKI 1+: 0.770; AKI 2+: 0.796; AKI 3+: 0.821) (full results in Table 2). Federated model stacking AUCs were approximately 0.03 lower than the pooled models (AKI 1+: 0.826; AKI 2+: 0.861; AKI 3+: 0.887), and these differences were attenuated by the inclusion of center-level metadata. Federated model stacking demonstrated comparable AUCs to weighted averaging.

**Table 2:** Temporal and external validation AUCs

Temporal validation			
	AKI-1 +	AKI-2 +	AKI-3 +
Base model^a^	0.7703 (0.7609, 0.7796)	0.7960 (0.7824, 0.8096)	0.8214 (0.8016, 0.8411)
Federated simple averaging	0.8292 (0.8221, 0.8363)	0.8542 (0.8448, 0.8635)	0.8375 (0.8221, 0.8528)
Federated weighted averaging	0.8411 (0.8343, 0.8479)	0.8699 (0.8614, 0.8785)	0.8836 (0.8715, 0.8956)
Federated model stacking (FMS)	0.8261 (0.8190, 0.8332)	0.8610 (0.8521, 0.8698)	0.8873 (0.8744, 0.9001)
Federated model stacking (FMS) with metadata^b^	0.8317 (0.8248, 0.8386)	0.8651 (0.8566, 0.8736)	0.8938 (0.8819, 0.9057)
Pooled model	0.8557 (0.8493, 0.8621)	0.8899 (0.8823, 0.8974)	0.9108 (0.9004, 0.9211)
External validation			
Base model	N/A	N/A	N/A
Federated simple averaging	0.8433 (0.8261, 0.8605)	0.8736 (0.8486, 0.8987)	0.8794 (0.8354, 0.9234)
Federated weighted averaging	0.8656 (0.8502, 0.881)	0.8948 (0.8747, 0.9149)	0.9288 (0.9009, 0.9567)
Federated model stacking (FMS)	0.8647 (0.8496, 0.8798)	0.9063 (0.8887, 0.924)	0.9333 (0.9040, 0.9626)
Federated model stacking (FMS) with metadata^c^	0.8623 (0.8472, 0.8774)	0.9012 (0.8829, 0.9196)	0.9312 (0.8994, 0.9631)
Pooled model	0.8824 (0.8685, 0.8964)	0.9249 (0.9087, 0.9412)	0.9496 (0.9287, 0.9705)

^a^Centers where a Base Model could not be trained are excluded from this temporal validation set.

^b^Metadata used: university affiliation, AKI stage 1 rate, AKI stage 2 rate and AKI stage 3 rate at each site.

^c^Metadata used: university affiliation.

In the external validation set, single-center model performance was not calculated because the external validation centers were excluded from the training set. The pooled models once again demonstrated the highest AUCs (AKI 1+: 0.882; AKI 2+: 0.925; AKI 3+: 0.950). The federated model AUCs were approximately 0.02 lower (AKI 1+: 0.865; AKI 2+: 0.906; AKI 3+: 0.933), and the inclusion of metadata did not substantially change the results. Federated model stacking again showed comparable performance to weighted averaging, and it slightly outperformed both averaging strategies for the more severe AKI outcomes (AKI-2+ and AKI-3+).

**Fig. 1:**
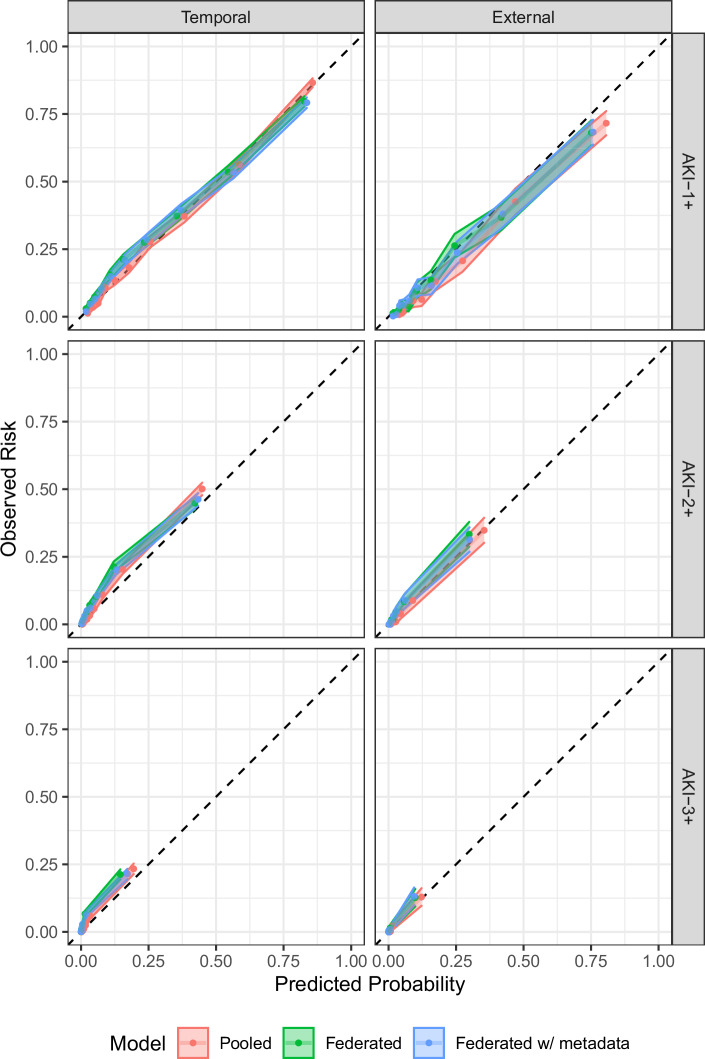
Calibration plot of the multicenter models. The calibration of the multicenter models in temporal validation and external validation, for all AKI severities. The predicted probabilities (deciles) are plotted against the observed probabilities with 95% confidence intervals. The diagonal line demonstrates the ideal calibration. The model calibration is examined for pooled model (red), federated model without center-level metadata (green), and federated model incorporating center-level metadata (blue).

The federated models, with and without metadata, and the pooled model were generally well-calibrated in both temporal and external validation for all AKI severities (Fig. 1). The first postoperative serum creatinine was the most important variable (Supplementary Fig. 5).

### Model performance at individual centers

While we found that the pooled model performs better in aggregate in both validation sets, not all centers may benefit equally from a multicenter modeling approach, either through pooling or federating. To examine the potential benefits to individual centers in building AI models as part of a network, we evaluated each hospital’s optimal modeling strategy using the temporal validation set for each center (Supplementary Fig. 2).

**Fig. 2:**
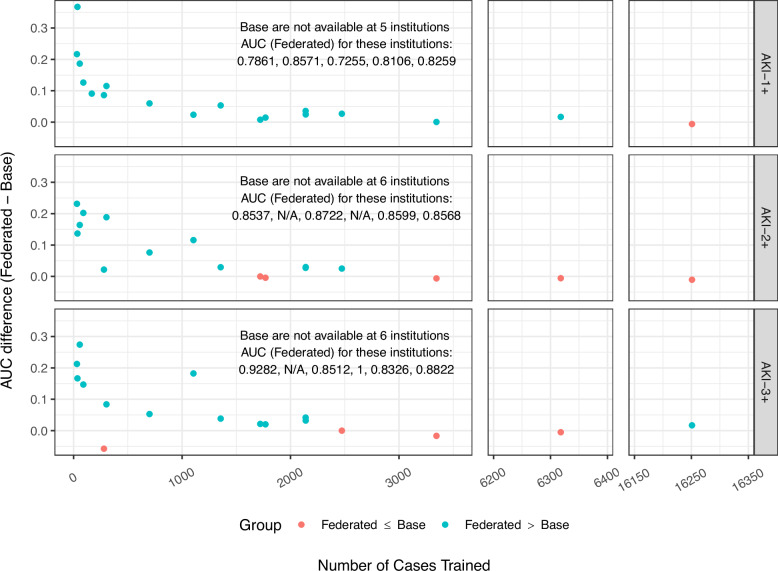
AUC differences between FMS and base models. Difference in AUC between federated (without metadata) and base models at each center in temporal validation, for all AKI severities. The better performing model is indicated by color (red: single-center base model, green: federated model).

Among the 23 centers in the validation set, using a single-center model was *not* the optimal strategy (based on the AUC point estimate) for *any* of the hospitals in predicting either AKI 1+, 2+, or 3+. Even if pooling data were not an option due to data sharing restrictions, single-center base models outperformed a simple federated approach (without metadata) for only 1 hospital for AKI 1+, 5 hospitals for AKI 2+, and 4 hospitals for AKI 1+ (Fig. 2). The single-center approach generally only performed better in the largest hospitals, and the magnitude of difference was small.

Five out of these 23 centers did not have a sufficient number of cases in the training set to even train a base model for AKI 1+ using the number of predictors in our model. Despite this limitation, 4 of these 5 centers achieved an AUC > 0.80 for AKI 1+ when pooling data, and 3 achieved an AUC > 0.80 when applying a federated learning approach.

### Network size and model performance

**Fig. 3:**
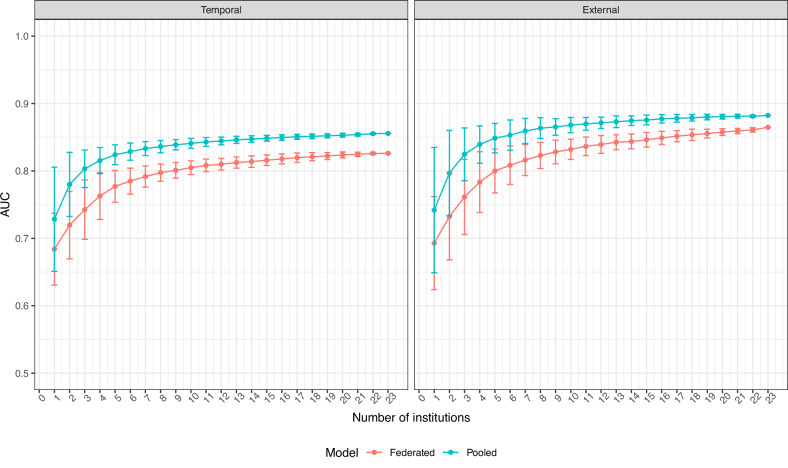
Learning curve of multicenter model performance in predicting AKI 1+ as network expands. Changes in model performance (AUC) in predicting AKI 1+ as the network size increases from none (no network) to 23 hospitals. Results from both temporal validation and external validation are shown. Different multicenter modeling approaches are compared (red: federated, green: pooled). For each network size, hospitals were randomly selected without replacement, and this process was repeated 100 times. The center dot and error bar at each network size represent the mean AUC and standard deviation, respectively, across 100 experiments.

While our results derive from a large national network of 31 hospitals, the value derived from building multicenter AI models may vary based on a network size. We found that the lowest AUC in both the temporal validation and external validation cohorts was for a network size of 1 (no network) and the highest AUC was for a network size of 23 (the full network available for study, Fig. 3). The AUC increased the most with the addition of the first few hospitals, and the magnitude became smaller as the final few hospitals were added to the network. About half of the increase in AUC from a single-center to the full 23-hospital model was observed with the model learning from only 4 hospitals.

## Discussion

In this retrospective study conducted using a national perioperative research network, we found that both multicenter modeling approaches produced a better model (substantially higher AUC) *in aggregate* for both the temporal and external validation sets as compared to local models. When examining the model performance at each individual center, a multicenter model had higher performance for *all* individual centers and *all* AKI outcomes versus a localized approach. This finding is particularly salient given the alternative conventional assumption that a single center’s models might be expected to perform better temporally on its own population without potential contamination by center-specific effects from other centers. We found that this conventional wisdom does not hold.

Even if data sharing were not an option, a federated model without hospital metadata outperformed a single-center modeling approach at the vast majority of hospitals for all outcomes, and the benefits of federating were the largest for hospitals with fewer cases. The benefits of building multicenter AI models were greatest for smaller networks, with diminishing returns observed with each additional hospital being added to the network.

Our study suggests that, contrary to the recent trend to build separate models for each hospital^3^, sharing data or model parameters across centers generally produces superior models than an approach relying on a bespoke model for each center. Even hospitals with high case volumes in our study obtained a higher-performing model from either pooling or federating, although this difference was marginal for the largest hospitals. On the other hand, the hospitals with the fewest training set cases either produced poorly performing local models or were unable to produce models due to a lack of sufficient events. Despite not contributing many cases to training, these hospitals generally saw high performance from pooled or federated models. Viewed through an equity lens, the centers with the largest case volume reap the smallest benefit from participating in an AI modeling research network, but their contribution of either data or base models to the research network greatly benefits the hospitals with smaller case volumes.

In this study, federated simple averaging and weighted averaging achieved performance comparable to federated model stacking, reflecting the relatively homogeneous nature of the participating hospitals, which are all acute care hospitals performing cardiac surgery. However, federated model stacking offers flexibility by allowing differential weighting of base model predictions and the incorporation of site-level metadata beyond sample size alone. This flexibility may be particularly valuable in networks with greater heterogeneity in practice patterns, patient populations, or institutional characteristics, where simple averaging approaches may be insufficient.

Recent work has explored federated stacking frameworks that share conceptual similarities with our approach. Cantu-Cervini et al.^19^ and Shaik et al.^20^ both propose federated learning strategies that rely on iterative communication until model convergence. In contrast, our federated model stacking is designed for one-round communication, which may be more practical in clinical settings where simplicity and scalability are critical. Additionally, our framework allows integration of center-level metadata into meta-model training, a feature not supported in prior work.

Our study has limitations. We only examined one clinical scenario (AKI), although we examined different severities of AKI with varying incidence and found largely concordant results regardless of the chosen severity of AKI outcome. While we did have a relatively large sample size, cardiac surgery occurs less commonly than conditions like sepsis or acute kidney injury among general inpatients. Thus, while our findings are robust to a relatively large sample size, our findings may not generalize to situations where individual centers can accumulate a much larger sample size than what we studied. Lastly, while we looked at calibration in the aggregate validation cohorts, we did not evaluate hospital-specific calibration. This will be important to consider in future work because multicenter AI models may need to be recalibrated for specific hospitals to ensure generalizability^21,22^.

Despite these limitations, our study has important implications for health systems considering whether the investment in research or quality networks focused on building multicenter AI models is sufficient to justify their participation. While prior evaluations of federated learning or pooling of data or coefficients have largely focused on aggregate performance from these approaches, we additionally show that the benefit to individual centers varies based on their sample size. The benefits are largest for centers with the smallest sample size and made possible by the participation of the larger centers. Thus, while the largest health systems may achieve comparable performance with a fully localized approach, their participation in multicenter efforts should be incentivized in the interest of population health, as it leads to direct benefits to smaller health systems.

## Methods

### Study design

We conducted a retrospective study using data from 31 hospitals within the Multicenter Perioperative Outcomes Group (MPOG) network, a national perioperative research and quality improvement network^23^, to examine the optimal modeling strategy for hospitals in the prediction of AKI. We compared a single-center predictive modeling strategy against two strategies that require participation in a research network—pooling data and federated learning.

The coordinating center of MPOG holds an institutional review board (IRB) approval (HUM00024166) that authorizes the establishment, data collection, and management of the centralized database. Each participating site obtains its own IRB approval to collect, organize, and submit a Limited Data Set to the coordinating center. These Limited Data Sets—containing no protected health information except for dates and extremes of age—are transmitted to coordinating center after rigorous efforts to ensure compliance^23^. A waiver of informed consent is obtained at each participating site.

We followed the Transparent Reporting of a multivariable prediction model for Individual Prognosis Or Diagnosis (TRIPOD) guidelines for conducting and reporting the findings from this study. Institutional review board approval (HUM00209313) was obtained for this observational study, and patient consent was waived. An a priori study protocol for inclusion criteria, data collection and handling, and statistical methods was approved and registered by the MPOG network’s Perioperative Clinical Research Committee.

### Study population

MPOG collects perioperative data from academic and community hospitals across 23 states in the United States. Methods for extraction of local electronic health record (EHR) data, validation, mapping to semantically interoperable concepts, and secure transfer to the MPOG data coordinating center have been previously described and used in multiple published studies.

Open cardiac surgical procedures using cardiopulmonary bypass performed on adult patients at US institutions from January 1, 2014, to February 1, 2022, were eligible for this study. Cases without preoperative (within 180 days) or postoperative (within 7 days) creatinine laboratory values, not meeting minimum data quality standards (see “Minimum data quality standards”), or from institutions contributing less than 20 cases annually meeting eligibility criteria, were also excluded. Finally, patients with pre-existing severe chronic kidney disease were excluded (Stage 4 or 5 based on an estimated glomerular filtration rate [eGFR] 15–29 or <15 mL/min/1.73m^2^, respectively); eGFR was computed using the baseline creatinine applied to the 2021 updated creatinine-based race-neutral equation. For patients undergoing repeated cardiac surgical procedures meeting the above inclusion criteria, only the index case was used.

### Minimum data quality standards

To ensure high data quality, each included case required a minimum data quality standard for inclusion in the dataset, defined as the presence of date of surgery, comorbidities, preoperative laboratory values, intraoperative arterial blood gas values, and physiologic monitoring data, baseline kidney function, and postoperative creatinine values (to derive CSA-AKI outcomes). Values of intraoperative time-varying variables were restricted to physiologically plausible, valid ranges.

### Predictor variables

We collected preoperative patient and surgical characteristics and time-varying intraoperative and immediate postoperative measures for each case. A full listing of the variables is provided in the Supplementary Table 3.

Patient characteristics included demographics, anthropometrics, comorbidities, preoperative laboratory values and vital signs, home medications, American Society of Anesthesiologists Physical Status classification, the baseline kidney function including eGFR and presence of preoperative AKI (as defined below), and the first postoperative serum creatinine^23^ (within 24 h). Surgical characteristics included emergent versus non-emergent, surgical procedure type (non-mutually exclusive), anesthesiology staffing model (presence of resident, nurse anesthetist, both, or neither with solely anesthesiology attending), weekday versus weekend start time, and academic versus community hospital.

Preoperative AKI was calculated by comparing the lowest baseline sCr value in the 60 days prior to surgery to the most recent serum creatinine value closest to surgery, staged for severity according to the Kidney Disease: Improving Global Outcomes (KDIGO) international guidelines: Stage 1 AKI was defined as a sCr level increase ≥ 0.3 mg/dL or ≥1.5 times baseline. Stage 2 AKI was defined as an increase of ≥2 times the baseline, and Stage 3 AKI ≥ 3 times baseline or an increase to ≥4.0 mg/dL.^27^

Intraoperative time-varying variables consisted of arterial blood gas values, physiologic monitors (systolic/mean/diastolic arterial blood pressure, central venous pressure, oxygen saturation, and heart rate), and intravenous cardiovascular medications administered intraoperatively (Supplementary Table 3). Given the dynamic nature of patient physiology surrounding initiation and separation from cardiopulmonary bypass (CPB), summary statistics for intraoperative variables were separately calculated as candidate predictors within models from each of three distinct phases: pre-CPB, intra-CPB, and post-CPB. More details can be found in Supplementary Table 3.

### Outcome: cardiac surgery associated AKI

AKI was defined based upon the maximum sCr level recorded between 2 and 7 days after the procedure. AKI was then defined and staged for severity according to the KDIGO international guidelines: no AKI, AKI stage 1, AKI stage 2, and AKI stage 3. While our models were trained using this multinomial outcome, results reported by AKI stages were grouped into binary outcomes according to the level of severity. For example, AKI stage 1+ (${P}_{{AKI}\ge 1}$) refers to any AKI stage (${P}_{{AKI}=1}+{P}_{{AKI}=2}+{P}_{{AKI}=3}$), and AKI stage 2+ (${P}_{{AKI}\ge 2}$) refers to AKI stage 2 and stage 3 (${P}_{{AKI}=2}+{P}_{{AKI}=3}$).

### Data split

After setting aside four centers for external validation (two randomly selected academic hospitals and two randomly selected community hospitals), we divided the remaining 27 institutions into a training set (January 1, 2014 to February 29, 2020) and a temporal validation set (March 1, 2020 to February 1, 2022) based on the timing of elective case scheduling changes induced by the COVID-19 pandemic. Because not all hospitals had eligible cases during the entire time period, some hospitals selected for temporal validation were only included in the training set or in the temporal validation set. A visual representation of the data split is shown in Supplementary Fig. 3.

### Development of single-center (base) models

To evaluate a single-center model approach, gradient-boosted decision tree (GBDT) models were separately trained and evaluated for each of the hospitals present in *both* the training and temporal validation sets. We opted for GBDT models because of their high empirical performance in prior applications to AKI prediction^7,24^. Details for the training and early stopping are presented in the **Gradient-Boosted Decision Tree Training and Early Stopping**. Centers in the external validation set were excluded from this evaluation.

### Development of pooled model

To assess the value of pooling data across multiple centers, we trained a pooled GBDT model in the training set and evaluated its performance in both the temporal and external validation cohorts. In contrast to the single-center models, the pooled model was evaluated in centers either absent from the training set entirely or with too few patients in the training set for a model to be adequately trained using data from that center only (*n* < 20 total cases, or no cases meeting an outcome definition).

The pooled model was trained using the combined training datasets from all participating sites, with full access to patient-level predictors and outcomes. As such, the pooled model represents the upper bound of achievable performance under full data sharing and serves as a performance ceiling for comparison with federated approaches.

### Development of federated models

We assessed the value of a federated learning approach by implementing a novel federated model stacking framework, which uses a two-stage training process based upon work developed for model stacking. In the first stage, base models are trained at each center and placed on a central server to be shared with all centers. In the second stage, predictions from all base models are used to train the final meta-model. A visual representation of the algorithm is shown in Supplementary Fig. 4a, b highlights which data are used in each training stage. In contrast to existing federated learning approaches, federated model stacking requires fewer rounds of model sharing across centers and is thus simpler to implement as centers are added or removed from the network.

### Federated model stacking algorithm details

In the federated model stacking algorithm, each center first partitions its data randomly into training, weighting, and testing (if model evaluation is desired) sets. During the first (base model building) stage, the following actions are taken: (a) each site uses its own training set to train a base model; (b) each site sends its base model to the central server; and (c) once the central server has all base models, it sends the collection of base models to all sites.

Upon receiving all base models, the second (meta-model building) stage starts and follows these steps: (a) each site applies all base models to its own weighting set to generate its weighting predictions; (b) each site sends its weighting predictions (one number per patient) to the central server; (c) the central server learns a meta model using the weighting predictions, and (d) once the meta model is learned, the central server sends the meta model back to all sites. The meta-model is used to weight the predictions generated by different base models.

Our framework also allows site-level metadata to be added when training the meta-model. The value remains the same as long as the cases are from the same site. When each site generates its weighting predictions, a column indicating whether the center is an academic hospital and three additional columns showing their rate of different AKI outcomes (AKI stage 1 rate, AKI stage 2 rate, and AKI stage 3 rate) calculated from the weighting data are added to the weighting prediction dataset and used to trained the meta-model.

The federated model stacking model with institutional-level metadata was trained using both university affiliation of the sites and site-level AKI rates. However, when it was evaluated on the external validation set, only university affiliation was available, and the AKI rates of the four held-out hospitals were set to missing.

### Federated model stacking pseudocode

Let:

${D}_{i}^{{train}}=\{({X}_{i}^{{train}},\,{y}_{i}^{{train}})\}$: training set at site $i$

${D}_{i}^{{weight}}=\{({X}_{i}^{{weight}},\,{y}_{i}^{{weight}})\}$: weighting set at site $i$

${D}_{i}^{{test}}=\{({X}_{i}^{{test}},\,{y}_{i}^{{test}})\}$: test set at site $i$

${f}_{i}$: base model trained at site $i$ using (${X}_{i}^{{train}},\,{y}_{i}^{{train}}$)

$F=\{{f}_{1},\,{f}_{2},\ldots ,\,{f}_{N}\}$: set of all base models across $N$ sites

${\hat{y}}_{{ij}}={f}_{j}({X}_{i}^{{weight}})$: predictions from base model $j$ applied to the weighting features at site $i$

${Z}_{i}=[{\hat{y}}_{i1},{\hat{y}}_{i2},\ldots {\hat{y}}_{{iN}}]$: prediction matrix at site $i$

$Z={\bigcup }_{i=1}^{N}{{\rm{Z}}}_{{\rm{i}}}$: aggregated prediction matrix across all sites

${y}^{{weight}}={\bigcup }_{i=1}^{N}{y}_{i}^{{weight}}$: aggregated weighting outcomes across all sites

$M$: optional site-level metadata (e.g., university affiliation, site-level AKI rates) associated with each row of $Z$

$g$: meta-model trained to map $(Z,M)$ to ${y}^{{weight}}$

Stage 1: Base Model Training

For each site $i\in \{1,\,\ldots ,{N}\}$:

1. Train a local model:
  $${f}_{i}={Train}({D}_{i}^{{train}})$$
2. Send ${f}_{i}$ to the central server.

The central server aggregates all base models $F$ and distributes $F$ back to each site.

Stage 2: Meta-Model Training

For each site $i$:

1. Generate weighting-site predictions using all base models:
  $${Z}_{i}={[\,f}_{i}\left({X}_{i}^{{weight}}\right),\,{f}_{2}\left({X}_{i}^{{weight}}\right),\ldots ,{f}_{N}\left({X}_{i}^{{weight}}\right)$$
2. Send ${Z}_{i}$ and ${y}_{i}^{{weight}}$ to the central server. (Optional: send site-level metadata ${M}_{i}$ if used.)
  At the central server:
3. Aggregate prediction matrices and outcomes:
  $$Z=\mathop{\bigcup }\limits_{i=1}^{N}{Z}_{i},\,{y}^{{weight}}=\,\mathop{\bigcup }\limits_{i=1}^{N}{y}_{i}^{{weight}}$$
4. Train a meta-model using predictions (and optional metadata) to predict the outcome:
  $$g={Train}(Z,\,{y}^{{weight}},\,M)$$
5. Distribute the meta-model $g$ to all sites for inference.

### Gradient-boosted decision tree training and early stopping

In our study, we used a gradient-boosted decision tree (GBDT) to train both the base models and the meta-model. We trained and compared two federated models: one using patient data only and one additionally incorporating center-level metadata.

GBDT were trained on a random 80% split of the training set to predict the outcome AKI stage as a multinomial outcome (i.e., “No AKI”, “AKI stage 1”, “AKI stage 2”, “AKI stage 3”, “AKI stage 3D”) for each case using 426 predictors with a maximum of 1000 trees and a maximum depth of 5. The remaining 20% of the training set was used to determine the need for early stopping based on an improvement in log loss lower than 0.0005 on 5 consecutive rounds based on a moving average calculated after every 10 trees. Categorical predictors were reordered by the mean response of each level for more efficient training. Internally, a separate one-versus-all tree was trained for each outcome class and averaged to produce probabilities for achieving each AKI stage postoperatively.

### Model evaluation

Model discrimination was assessed using the area under the receiver operating characteristic curve (AUC). A separate AUC was reported for individuals at risk for each AKI stage. For example, patients without any AKI prior to surgery were evaluated on their risk of developing any AKI (i.e., stage 1 or greater), and patients with no AKI or AKI stage 1 were evaluated on their risk of developing AKI stage 2 or greater, and so on. The 95% confidence intervals were generated using DeLong’s method. Model calibration was evaluated by comparing deciles of predicted probabilities with observed risks for temporal and external validation sets, for all AKI outcome severities.

Models were evaluated in aggregate (across all centers in both validation sets) and then individually for each hospital. For each hospital, we determined the optimal modeling strategy by comparing the performance of single-center (base) models against pooled and federated models. The individual hospital analysis was performed using the temporal validation set only because there were no base models available for use in the external validation sets.

### Sensitivity analysis

To assess the robustness of federating approach, we conducted a sensitivity analysis with two other federated aggregation strategies: simple averaging and weighted averaging. For simple averaging, we computed the final prediction for each encounter by taking the mean value of predictions generated by all base models across participating centers. For weighted averaging, we calculated a weighted mean of the base model predictions for each encounter, where the weight assigned to each center’s prediction was proportional to its training sample size. The results of this empirical comparison are presented in Table 2.

### Studying the role of network size in resulting model performance

Building AI models as part of a research network may involve more investment as the network expands, but larger networks may produce more stable and generalizable models due to greater sample size and diversity. To examine the role of network size on model performance, we performed a learning curve analysis in which we compared the performance of pooled and federated models predicting AKI 1+ by varying the size of the network from 1 hospital (no network) up to 23 hospitals. For each network size, hospitals were randomly selected without replacement, and this process was repeated 100 times.

### Feature importance

To develop an understanding of which variables most strongly contributed to the predictive performance of models developed, evaluated the feature importance. Feature importance of variables within the pooled model was assessed using each feature’s squared influence within the GBDT algorithm aggregated over the tree ensemble. Feature importance for the pooled model is provided in Supplementary Fig. 5.

### Software

All data processing and analyses were performed using R 4.2.1. Transformation of time-series data and calculation of summary statistics were performed using the Grammar of Prediction (gpmodels) R package. h2o version 3.38.0.1 was used to fit all GBDT models, including the pooled model, base models, and the meta model of the federated model. Figure 2 with axis breaks was prepared using the ggbreak R package^25,26^.

## Supplementary information

Supplementary Information – clean

## Source data

Source Data Figure 2 and Supplementary Figure 2

Source Data Figure 3

## Data Availability

The datasets involved in this study are defined as limited datasets per United States Federal Regulations and require execution of a data use agreement for transfer or use of the data. They are derived from data shared within the Multicenter Perioperative Outcomes Group (MPOG). The investigative team is able to share data securely and transparently conditional on: (i) receipt of a detailed written request identifying the requestor, purpose and proposed use of the shared data, (ii) use of a secure enclave for the sharing of personally identifiable information and (iii) the request is permissible within the confines of existing data use agreements executed between MPOG members.

## Code availability

The code used in this study is available from the corresponding author upon request.
