# Supplementary material for: Exploring the limits of localization: federated model stacking improves hospital-level prediction in a national research network
Source: NPJ Digit Med. 2026 Apr 24;9:492. doi: 10.1038/s41746-026-02634-1 (PMC13314945; doi:10.1038/s41746-026-02634-1)
Supplement: Supplementary file 1 — Supplementary Information – clean [file 41746_2026_2634_MOESM1_ESM.pdf]

SUPPLEMENTARY INFORMATION

Supplementary Figure 1. Study Flow Diagram 2

Supplementary Figure 2. AUC comparison of base, federated and pooled models for temporal validation set 3

Supplementary Figure 3. Visual representation of study data split 4

Supplementary Figure 4. Visual representation of the federated model stacking algorithm 5

Supplementary Figure 5. Feature importance plot of the pooled model 7

Supplementary Table 1. Number of cases at each site by data partition 8

Supplementary Table 2. Extended patient and surgical characteristics 9

Supplementary Table 3. Description of variables and predictors in the study models 15

Supplementary Figure 1. Study Flow Diagram

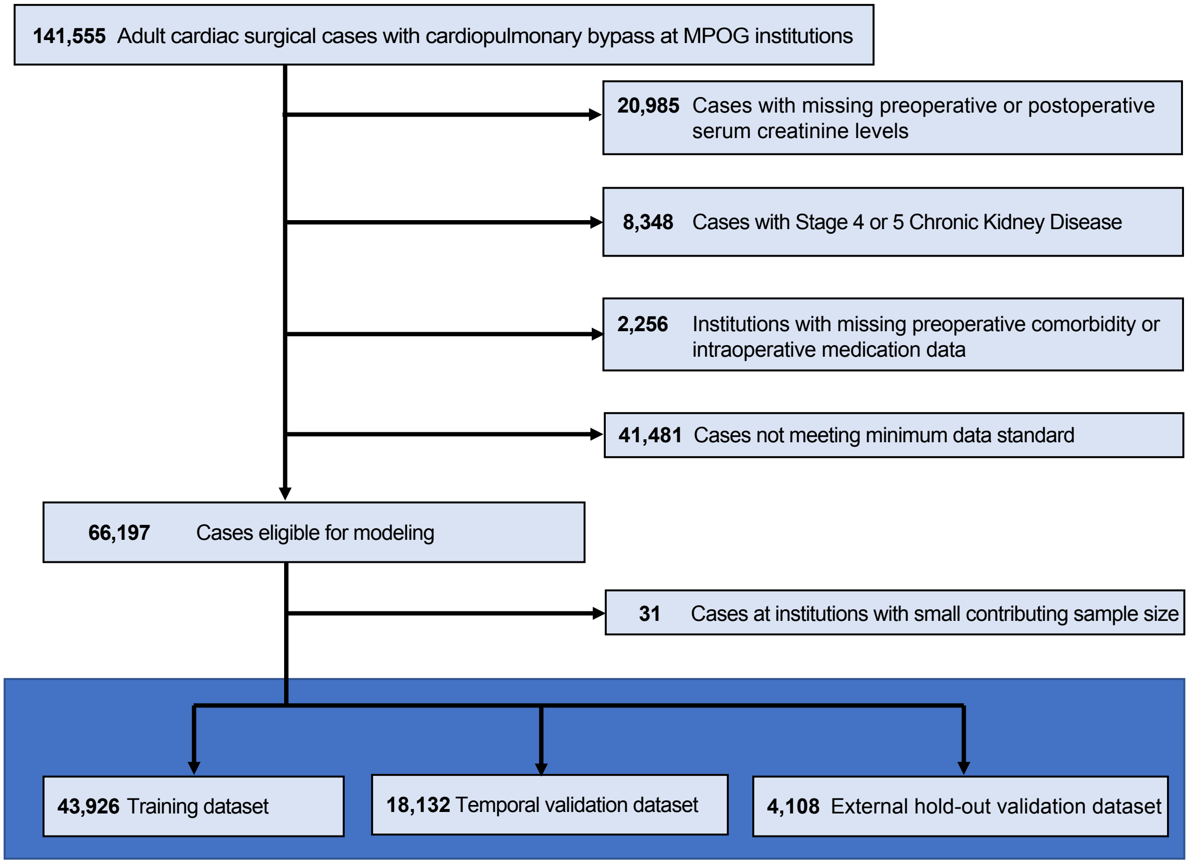

The flow diagram shows the case inclusion and exclusion criteria for the analysis of the study. Number of remaining cases after each step are shown. Numbers of cases included in each data split for subsequent training and evaluation are also shown.

Supplementary Figure 2. AUC comparison of base, federated and pooled models for temporal validation set

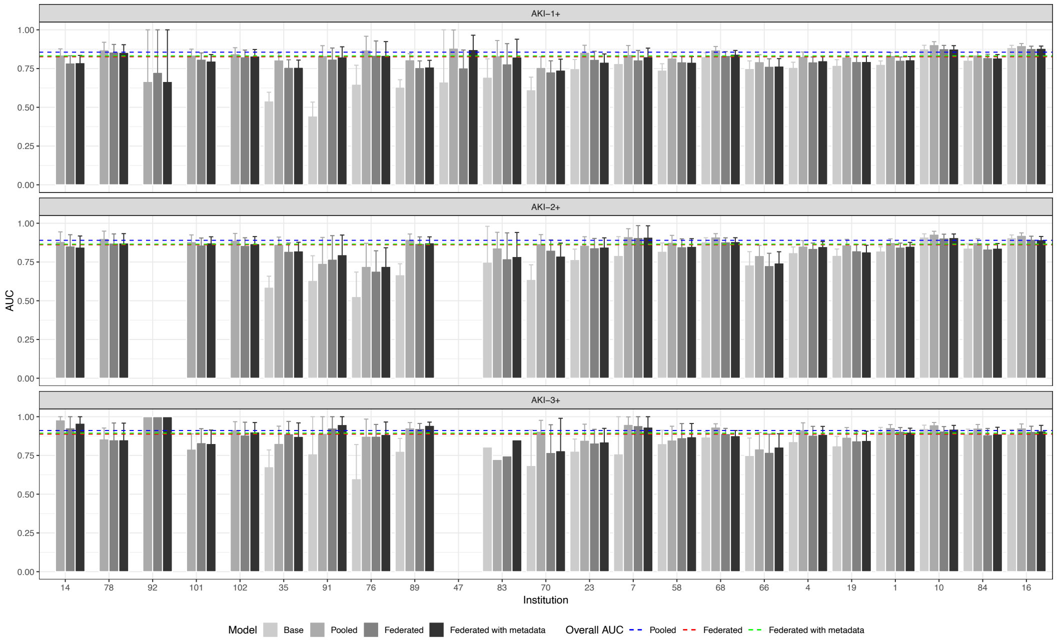

Outcome	Model	Overall	14	78	92	101	102	35	91	76	89	47	83	70	23	7	58	68	66	4	19	1	10	84	16
			Nt = 0	Nt = 0	Nt = 0	Nt = 0	Nt = 15	Nt = 31	Nt = 36	Nt = 57	Nt = 90	Nt = 168	Nt = 280	Nt = 303	Nt = 700	Nt = 1105	Nt = 1356	Nt = 1721	Nt = 1769	Nt = 2139	Nt = 2140	Nt = 2473	Nt = 3345	Nt = 6318	Nt = 16251
			Nv = 744	Nv = 370	Nv = 20	Nv = 548	Nv = 473	Nv = 521	Nv = 275	Nv = 94	Nv = 724	Nv = 58	Nv = 88	Nv = 205	Nv = 326	Nv = 266	Nv = 918	Nv = 1374	Nv = 560	Nv = 972	Nv = 825	Nv = 1956	Nv = 1403	Nv = 1941	Nv = 3469
AKI−1+	Base	NA	NA	NA	NA	NA	NA	0.5412	0.4440	0.6492	0.6303	0.6636	0.6944	0.6134	0.7497	0.7825	0.7402	0.8268	0.7503	0.7576	0.7708	0.7771	0.8778	0.8040	0.8857
	Pooled	0.8557	0.8377	0.8710	0.6667	0.8369	0.8459	0.8057	0.8347	0.8702	0.8068	0.8818	0.8328	0.7573	0.8552	0.8407	0.8186	0.8703	0.7945	0.8282	0.8245	0.8347	0.9045	0.8378	0.8982
	Federated	0.8261	0.7861	0.8571	0.7255	0.8106	0.8259	0.7579	0.8113	0.8357	0.7565	0.7545	0.7805	0.7285	0.8095	0.8061	0.7935	0.8346	0.7648	0.7933	0.7954	0.8039	0.8784	0.8208	0.8797
	Federated with metadata	0.8317	0.7881	0.8543	0.6667	0.7986	0.8311	0.7581	0.8252	0.8351	0.7602	0.8727	0.8246	0.7403	0.7912	0.8262	0.7910	0.8428	0.7663	0.8007	0.7956	0.8068	0.8764	0.8185	0.8805
AKI−2+	Base	NA	NA	NA	NA	NA	NA	0.5876	0.6315	0.5271	0.6678	NA	0.7494	0.6377	0.7658	0.7923	0.8193	0.8805	0.7313	0.8104	0.7927	0.8212	0.9114	0.8396	0.9071
	Pooled	0.8899	0.8828	0.9022	NA	0.8813	0.8899	0.8640	0.7414	0.7216	0.8967	NA	0.8410	0.8683	0.8581	0.9139	0.8774	0.9119	0.7917	0.8527	0.8608	0.8758	0.9298	0.8754	0.9219
	Federated	0.8610	0.8537	0.8722	NA	0.8599	0.8568	0.8190	0.7682	0.6910	0.8701	NA	0.7711	0.8262	0.8420	0.9080	0.8486	0.8803	0.7270	0.8377	0.8225	0.8462	0.9050	0.8339	0.8965
	Federated with metadata	0.8651	0.8464	0.8730	NA	0.8737	0.8685	0.8218	0.7973	0.7216	0.8742	NA	0.7855	0.7884	0.8466	0.9104	0.8509	0.8816	0.7440	0.8497	0.8154	0.8516	0.9076	0.8393	0.8944
AKI−3+	Base	NA	NA	NA	NA	NA	NA	0.6774	0.7605	0.6000	0.7770	NA	0.8046	0.6851	0.7776	0.7602	0.8263	0.8696	0.7499	0.8393	0.8121	0.9060	0.9243	0.8887	0.8879
	Pooled	0.9108	0.9796	0.8568	1	0.7910	0.9164	0.8276	0.8926	0.8742	0.9281	NA	0.7241	0.9058	0.8480	0.9502	0.8499	0.9333	0.7923	0.9156	0.8678	0.9302	0.9479	0.9268	0.9280
	Federated	0.8873	0.9282	0.8512	1	0.8326	0.8822	0.8900	0.9272	0.8742	0.9240	NA	0.7471	0.7690	0.8305	0.9425	0.8647	0.8911	0.7701	0.8810	0.8443	0.9060	0.9077	0.8837	0.9049
	Federated with metadata	0.8938	0.9581	0.8513	1	0.8271	0.9020	0.8729	0.9494	0.8854	0.9440	NA	0.8506	0.7807	0.8374	0.9333	0.8709	0.8778	0.8044	0.8859	0.8469	0.9009	0.9200	0.8890	0.9087

Temporal validation AUC comparison of 4 models (base, federated model stacking, federated model stacking with metadata, and pooled) at each individual institution (AKI-1+, AKI-2+, AKI-3+). Source data for this figure is provided in Source Data – Supplementary Figure 2.xlsx.

Supplementary Figure 3. Visual representation of study data split

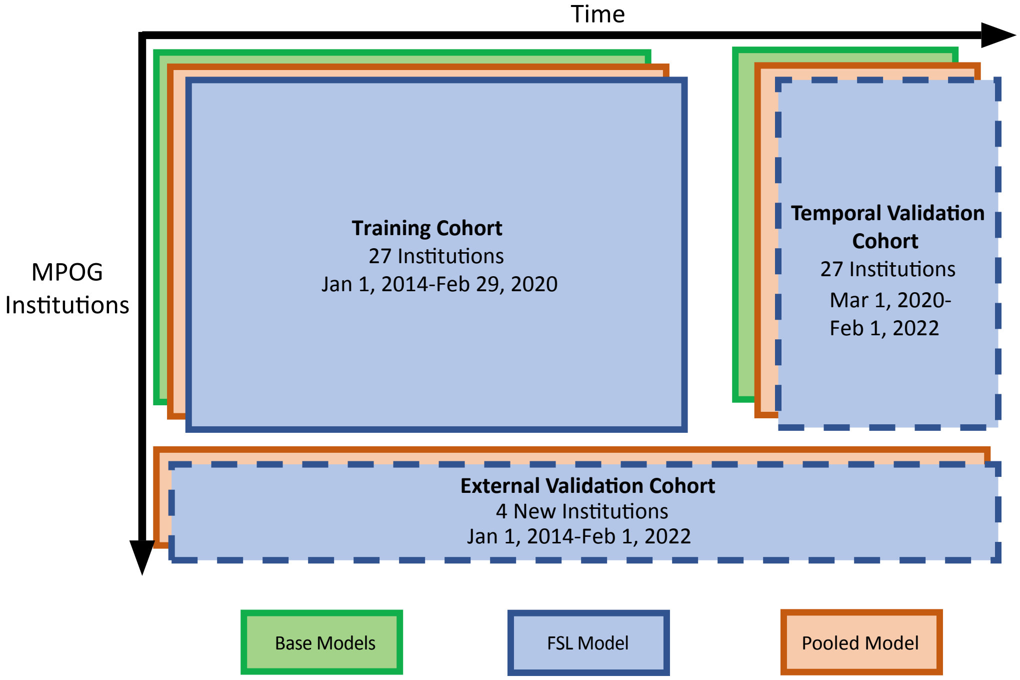

Among all 31 centers participated in the study, four centers were set aside for external validation. The remaining 27 centers were split into a training set and a temporal validation set based on the timing of elective case scheduling changes induced by the COVID-19 pandemic. Single-center (base) models and multicenter models (pooled and federated) were trained using the training set. Base models were only tested on temporal validation set, while pooled and federated models were tested on both temporal validation and external validation sets.

Supplementary Figure 4. Visual representation of the federated model stacking algorithm

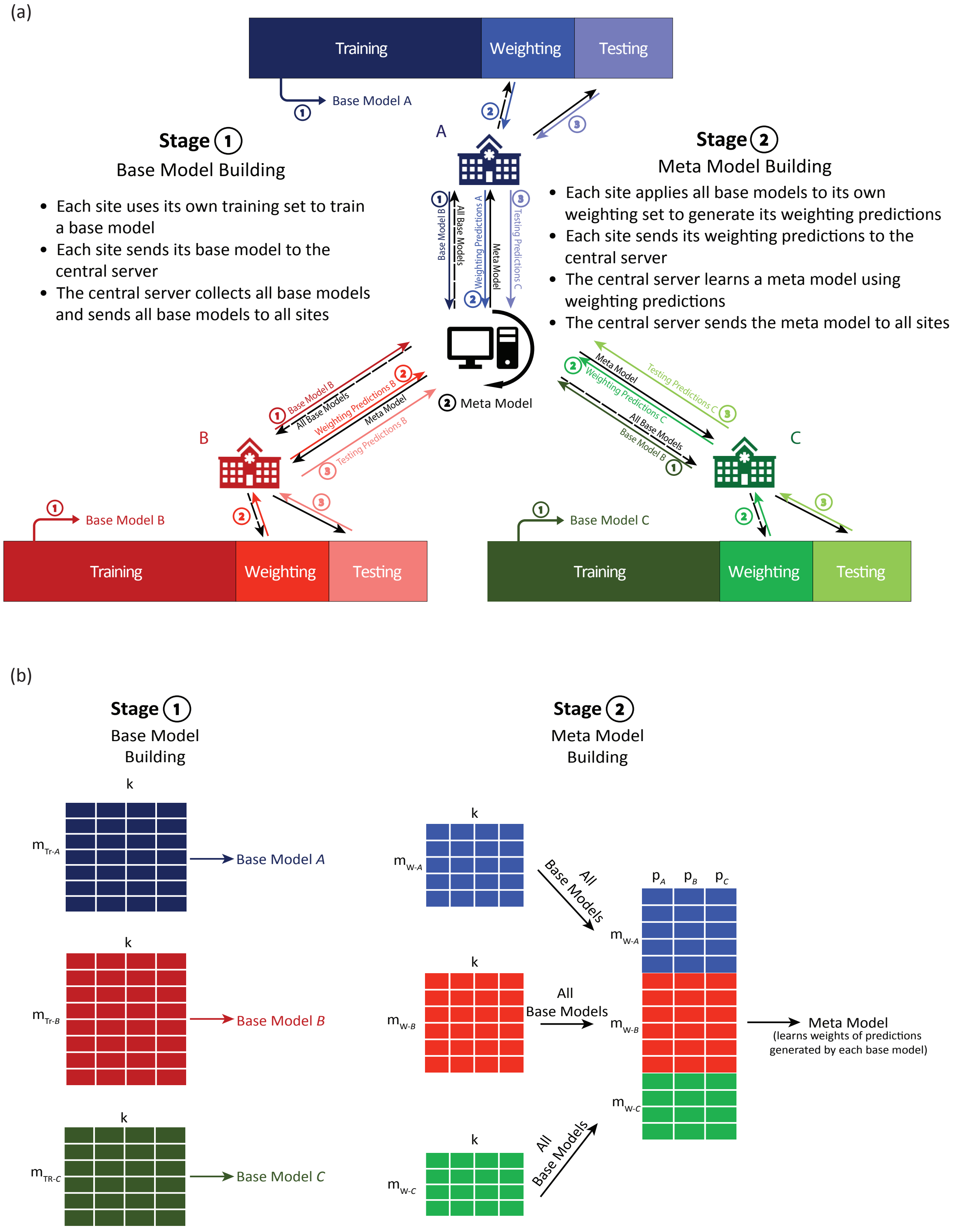

(a) Data exchanges between centers associated with each stage of federated model stacking algorithm. (b) Changes of data structure used in each stage of federated model stacking algorithm.

Supplementary Figure 5. Feature importance plot of the pooled model

Top 20 important features of the pooled model. Predictors are ranked by their relative importance and expressed as a percentage.

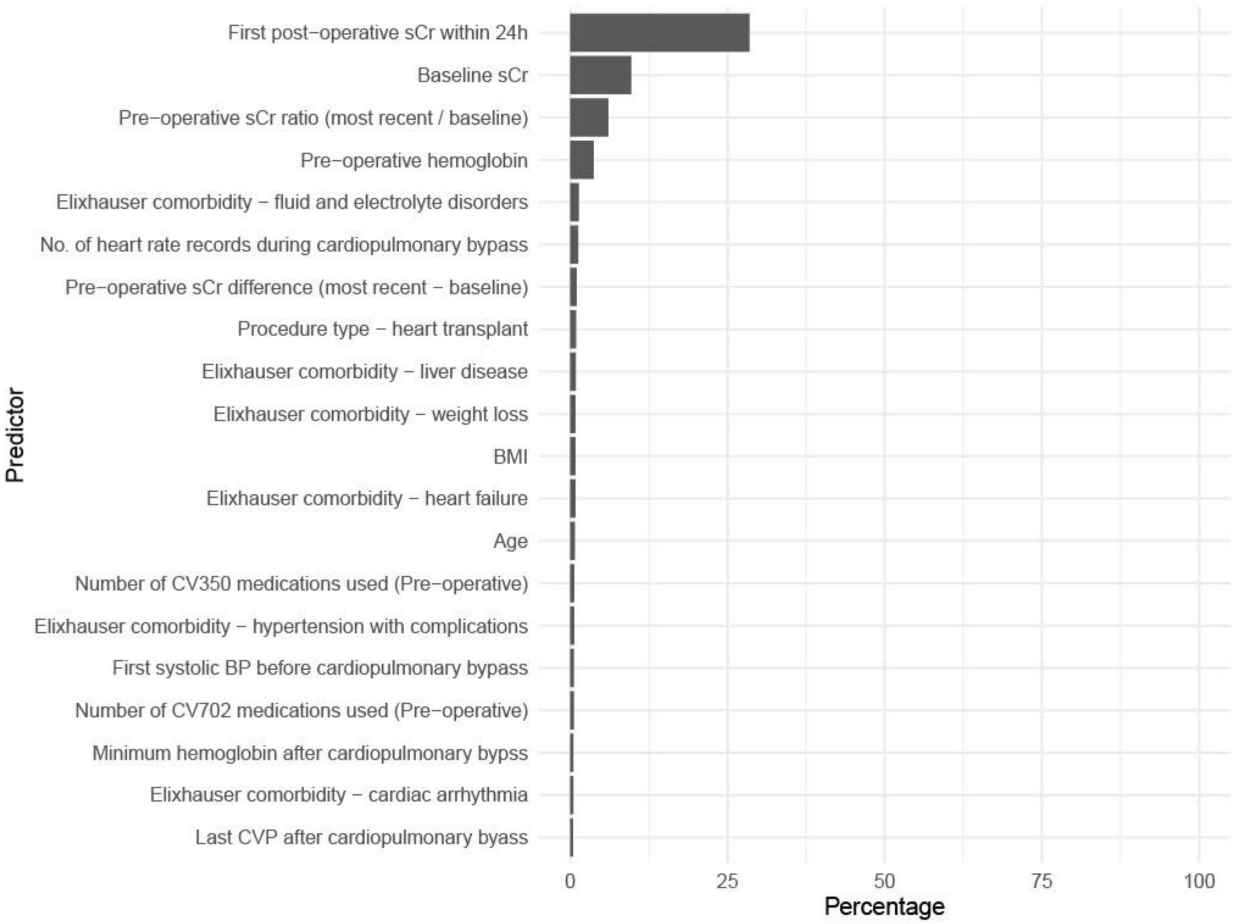

Supplementary Table 1. Number of cases at each site by data partition

Institution	Training	Temporal Validation	External Validation	University-affiliated/Academic
1	2,473	1,956	-	Yes
4	2,139	972	-	Yes
5	1,811	0	-	Yes
7	1,105	266	-	Yes
10	3,345	1,403	-	Yes
14	0	744	-	Yes
16	16,251	3,469	-	Yes
19	2,140	825	-	Yes
23	700	326	-	Yes
32	-	-	238	No
35	31	521	-	Yes
37	-	-	989	Yes
38	829	1	-	Yes
40	728	1	-	No
46	-	-	259	No
47	168	58	-	Yes
58	1,356	918	-	Yes
65	261	0	-	Yes
66	1,769	560	-	Yes
68	1,721	1,374	-	Yes
70	303	205	-	Yes
76	57	94	-	Yes
78	0	370	-	Yes
83	280	88	-	Yes
84	6,318	1,941	-	Yes
86	-	-	2,622	Yes
89	90	724	-	Yes
91	36	275	-	Yes
92	0	20	-	No
101	0	548	-	Yes
102	15	473	-	Yes
**Total**	43,926	18,132	4,108	

Supplementary Table 2. Extended patient and surgical characteristics

Characteristic		Overall (N = 66,166)	Training (N = 43,926)	Temporal Validation (N = 18,132)	External Validation (N = 4,108)
**Preoperative Patient Characteristics**					
**Age (years)**		62.0 (13.5)	61.9 (13.6)	61.7 (13.3)	64.4 (12.3)
**Sex**					
	Female	20,921 (31.6%)	14,062 (32.0%)	5,726 (31.6%)	1,133 (27.6%)
	Male	45,245 (68.4%)	29,864 (68.0%)	12,406 (68.4%)	2,975 (72.4%)
**Race / Ethnicity**					
	White not of hispanic origin	52,643 (79.6%)	34,822 (79.3%)	14,335 (79.1%)	3,486 (84.9%)
	Black not of hispanic origin	4,304 (6.5%)	2,720 (6.2%)	1,494 (8.2%)	90 (2.2%)
	Asian or Pacific Islander	2,068 (3.1%)	1,215 (2.8%)	624 (3.4%)	229 (5.6%)
	Bi or Multi Racial	569 (0.9%)	413 (0.9%)	156 (0.9%)	0 (0.0%)
	American Indian or Alaska Native	180 (0.3%)	102 (0.2%)	66 (0.4%)	12 (0.3%)
	Hispanic white	514 (0.8%)	267 (0.6%)	206 (1.1%)	41 (1.0%)
	Hispanic black	38 (0.1%)	18 (0.0%)	19 (0.1%)	1 (0.0%)
	Middle Eastern	38 (0.1%)	38 (0.1%)	0 (0.0%)	0 (0.0%)
	Missing	5,812 (8.8%)	4,331 (9.9%)	1,232 (6.8%)	249 (6.1%)
**Height (cm)**		171.9 (14.1)	172.5 (10.9)	170.2 (19.9)	172.4 (10.4)
	Missing	3,424 (5.2%)	2,877 (6.5%)	524 (2.9%)	23 (0.6%)
**Weight (kg)**		87.0 (20.8)	86.7 (20.7)	87.7 (21.2)	87.2 (21.0)
	Missing	1,795 (2.7%)	1,055 (2.4%)	734 (4.0%)	6 (0.1%)
**Body Mass Index (kg/m^2)**		29.1 (6.2)	29.0 (6.2)	29.2 (6.3)	29.3 (6.3)
	Missing	4,672 (7.1%)	3,071 (7.0%)	1,572 (8.7%)	29 (0.7%)

**Smoking Classification**					
	Non-Smoker	3,450 (21.3%)	2,074 (18.6%)	1,106 (29.1%)	270 (21.6%)
	Smoker	5,232 (32.3%)	3,705 (33.3%)	1,198 (31.5%)	329 (26.3%)
	Former Smoker	7,017 (43.3%)	5,074 (45.6%)	1,301 (34.2%)	642 (51.3%)
	Conflicting Documentation	489 (3.0%)	283 (2.5%)	196 (5.2%)	10 (0.8%)
**ASA Physical Status Classification**					
	ASA Class 1	59 (0.1%)	54 (0.1%)	3 (0.0%)	2 (0.0%)
	ASA Class 2	397 (0.6%)	267 (0.6%)	72 (0.4%)	58 (1.4%)
	ASA Class 3	13,838 (20.9%)	8,806 (20.0%)	3,130 (17.3%)	1,902 (46.3%)
	ASA Class 4	50,982 (77.1%)	34,286 (78.1%)	14,614 (80.6%)	2,082 (50.7%)
	ASA Class 5	890 (1.3%)	513 (1.2%)	313 (1.7%)	64 (1.6%)
**Preoperative Laboratory Values**					
**Platelet Count, (K/mL)**		218.6 (72.2)	216.9 (70.2)	221.9 (77.2)	223.0 (70.4)
	Missing	1,122 (1.7%)	134 (0.3%)	984 (5.4%)	4 (0.1%)
**White Blood Cell Count (per mL)**		7.6 (3.1)	7.5 (3.1)	7.7 (3.1)	7.9 (3.4)
	Missing	1,077 (1.6%)	1,058 (2.4%)	17 (0.1%)	2 (0.0%)
**Sodium (mEq/L)**		138.8 (3.2)	139.1 (3.2)	138.3 (3.1)	138.5 (3.1)
**Potassium (mEq/L)**		4.2 (0.4)	4.2 (0.4)	4.2 (0.4)	4.1 (0.4)
**Glucose (g/dL)**		115.8 (39.3)	115.1 (39.6)	116.5 (38.7)	119.0 (38.0)
**Hemoglobin (g/dL)**		13.3 (2.0)	13.3 (2.0)	13.2 (2.1)	13.3 (1.9)
**Bicarbonate (mmol/L)**		25.6 (3.1)	25.8 (3.1)	25.3 (3.3)	24.8 (2.8)
**Creatinine-Related Variables**					
**Preoperative Baseline Serum Creatinine, g/dL**		1.0 (0.3)	1.0 (0.3)	0.9 (0.3)	0.9 (0.3)
**Preoperative Most Recent Serum Creatinine, g/dL**		1.0 (0.5)	1.0 (0.6)	1.0 (0.3)	1.0 (0.3)

**Preoperative Serum Creatinine Ratio (Most Recent/Baseline)**	1.1 (0.5)	1.1 (0.6)	1.1 (0.2)	1.1 (0.2)
**Preoperative Serum Creatinine Difference (Most Recent - Baseline)**	0.1 (0.5)	0.1 (0.5)	0.1 (0.1)	0.1 (0.1)
**First Post-operative Serum Creatinine Within 24h**	1.0 (0.3)	1.0 (0.3)	1.0 (0.3)	0.9 (0.3)
Missing	286 (0.4%)	222 (0.5%)	60 (0.3%)	4 (0.1%)
**Preoperative AKI**				
No Preoperative AKI	62,831 (95.0%)	41,831 (95.2%)	17,081 (94.2%)	3,919 (95.4%)
Preoperative AKI-1	3,066 (4.6%)	1,934 (4.4%)	967 (5.3%)	165 (4.0%)
Preoperative AKI-2	218 (0.3%)	136 (0.3%)	66 (0.4%)	16 (0.4%)
Preoperative AKI-3	51 (0.1%)	25 (0.1%)	18 (0.1%)	8 (0.2%)
**Preoperative Patient Comorbidities (Elixhauser)**				
**AIDS/HIV**	195 (0.3%)	110 (0.3%)	57 (0.3%)	28 (0.7%)
**Alcohol Abuse**	619 (0.9%)	497 (1.1%)	94 (0.5%)	28 (0.7%)
**Blood Loss Anemia**	1,532 (2.3%)	1,053 (2.4%)	398 (2.2%)	81 (2.0%)
**Cardiac Arrhythmia**	42,934 (64.9%)	27,245 (62.0%)	12,912 (71.2%)	2,777 (67.6%)
**Chronic Pulmonary Disease**	15,116 (22.8%)	10,143 (23.1%)	4,008 (22.1%)	965 (23.5%)
**Coagulopathy**	25,473 (38.5%)	15,989 (36.4%)	8,898 (49.1%)	586 (14.3%)
**Congestive Heart Failure**	31,495 (47.6%)	19,952 (45.4%)	9,910 (54.7%)	1,633 (39.8%)
**Deficiency Anemia**	3,142 (4.7%)	1,925 (4.4%)	1,040 (5.7%)	177 (4.3%)
**Depression**	9,898 (15.0%)	6,071 (13.8%)	3,160 (17.4%)	667 (16.2%)
**Diabetes with Complications**	6,719 (10.2%)	4,158 (9.5%)	2,159 (11.9%)	402 (9.8%)
**Diabetes without Complications**				
No	54,197 (81.9%)	36,329 (82.7%)	14,946 (82.4%)	2,922 (71.1%)
Yes	11,851 (17.9%)	7,503 (17.1%)	3,162 (17.4%)	1,186 (28.9%)
Missing	118 (0.2%)	94 (0.2%)	24 (0.1%)	0 (0.0%)
**Drug Abuse**	2,672 (4.0%)	1,559 (3.5%)	888 (4.9%)	225 (5.5%)

**Fluid and Electrolyte Disorders**		39,655 (59.9%)	25,740 (58.6%)	12,602 (69.5%)	1,313 (32.0%)
**Hypertension**		24,791 (37.5%)	14,337 (32.6%)	8,981 (49.5%)	1,473 (35.9%)
**Hypothyroidism**		9,223 (13.9%)	6,235 (14.2%)	2,507 (13.8%)	481 (11.7%)
**Liver Disease**		4,758 (7.2%)	2,850 (6.5%)	1,609 (8.9%)	299 (7.3%)
**Lymphoma**		496 (0.7%)	335 (0.8%)	124 (0.7%)	37 (0.9%)
**Metastatic Cancer**		346 (0.5%)	218 (0.5%)	105 (0.6%)	23 (0.6%)
**Obesity**		16,562 (25.0%)	10,161 (23.1%)	5,387 (29.7%)	1,014 (24.7%)
**Other Neurological Disorders**		5,337 (8.1%)	3,181 (7.2%)	1,790 (9.9%)	366 (8.9%)
**Paralysis**		1,267 (1.9%)	788 (1.8%)	394 (2.2%)	85 (2.1%)
**Peptic Ulcer Disease excluding Bleeding**		628 (0.9%)	413 (0.9%)	183 (1.0%)	32 (0.8%)
**Peripheral Vascular Disorders**		24,588 (37.2%)	15,781 (35.9%)	7,557 (41.7%)	1,250 (30.4%)
**Psychoses**		470 (0.7%)	303 (0.7%)	137 (0.8%)	30 (0.7%)
**Pulmonary Circulation Disorders**		12,034 (18.2%)	7,682 (17.5%)	3,632 (20.0%)	720 (17.5%)
**Rheumatoid Arthritis / Collagen Vascular Disease**					
	No	63,888 (96.6%)	42,446 (96.6%)	17,463 (96.3%)	3,979 (96.9%)
	Yes	2,160 (3.3%)	1,386 (3.2%)	645 (3.6%)	129 (3.1%)
	Missing	118 (0.2%)	94 (0.2%)	24 (0.1%)	0 (0.0%)
**Solid Tumor without Metastatis**					
	No	64,437 (97.4%)	42,830 (97.5%)	17,616 (97.2%)	3,991 (97.2%)
	Yes	1,611 (2.4%)	1,002 (2.3%)	492 (2.7%)	117 (2.8%)
	Missing	118 (0.2%)	94 (0.2%)	24 (0.1%)	0 (0.0%)
**Valvular Disease**					
	Yes	46,092 (69.7%)	31,145 (70.9%)	12,513 (69.0%)	2,434 (59.3%)
	No	19,956 (30.2%)	12,687 (28.9%)	5,595 (30.9%)	1,674 (40.7%)
	Missing	118 (0.2%)	94 (0.2%)	24 (0.1%)	0 (0.0%)

**Weight Loss**					
	No	60,259 (91.1%)	40,069 (91.2%)	16,286 (89.8%)	3,904 (95.0%)
	Yes	5,789 (8.7%)	3,763 (8.6%)	1,822 (10.0%)	204 (5.0%)
	Missing	118 (0.2%)	94 (0.2%)	24 (0.1%)	0 (0.0%)
**Surgical Characteristics - Procedure Type**					
**Valve Only**		21,670 (32.8%)	15,371 (35.0%)	5,232 (28.9%)	1,067 (26.0%)
**Coronary Artery Bypass Only**		18,573 (28.1%)	11,350 (25.8%)	5,356 (29.5%)	1,867 (45.4%)
**Aortic**		9,316 (14.1%)	6,114 (13.9%)	2,852 (15.7%)	350 (8.5%)
**Valve + Coronary Artery Bypass Only**		6,455 (9.8%)	4,433 (10.1%)	1,492 (8.2%)	530 (12.9%)
**Myectomy**		2,156 (3.3%)	1,588 (3.6%)	541 (3.0%)	27 (0.7%)
**Ventricular Assist Device**		1,680 (2.5%)	1,161 (2.6%)	463 (2.6%)	56 (1.4%)
**Heart Transplant**		1,669 (2.5%)	953 (2.2%)	664 (3.7%)	52 (1.3%)
**Pulmonary Thromboendarterectomy**		385 (0.6%)	242 (0.6%)	143 (0.8%)	0 (0.0%)
**Other**		4,264 (6.4%)	2,716 (6.2%)	1,389 (7.7%)	159 (3.9%)
**Additional Surgical Characteristics**					
**Anesthesia Duration (min)**		419 (133)	417 (133)	434 (134)	371 (121)
**Cardiopulmonary Bypass Duration (min)**		140 (88)	133 (85)	157 (99)	137 (65)
**Circulatory Arrest Used**		1,267 (1.9%)	790 (1.8%)	464 (2.6%)	13 (0.3%)
**Intra-Aortic Balloon Pump Used**		843 (1.3%)	452 (1.0%)	367 (2.0%)	24 (0.6%)
**Other Mechanical Support Used following CPB**		226 (0.3%)	106 (0.2%)	118 (0.7%)	2 (0.0%)
**Emergent**					
	No	61,605 (93.1%)	41,313 (94.1%)	16,465 (90.8%)	3,827 (93.2%)
	Yes	4,059 (6.1%)	2,613 (5.9%)	1,165 (6.4%)	281 (6.8%)
	Missing	502 (0.8%)	0 (0.0%)	502 (2.8%)	0 (0.0%)
**Anesthesia Technique**					
	General - ETT	65,097 (98.4%)	43,225 (98.4%)	17,771 (98.0%)	4,101 (99.8%)

General - LMA followed by ETT	1,069 (1.6%)	701 (1.6%)	361 (2.0%)	7 (0.2%)
**Case Scheduling / Staffing Characteristics**				
**Weekend**				
Weekday	64,317 (97.2%)	42,829 (97.5%)	17,498 (96.5%)	3,990 (97.1%)
Weekend	1,849 (2.8%)	1,097 (2.5%)	634 (3.5%)	118 (2.9%)
**Holiday**	240 (0.4%)	144 (0.3%)	79 (0.4%)	17 (0.4%)
**Anesthesiology Resident Present ***	44,045 (66.6%)	28,972 (66.0%)	11,801 (65.1%)	3,272 (79.6%)
**Nurse Anesthetist Present ***	13,117 (19.8%)	9,668 (22.0%)	3,060 (16.9%)	389 (9.5%)
**Anesthesiology Attending Only**	9,085 (13.7%)	5,320 (12.1%)	3,313 (18.3%)	452 (11.0%)
**Institutional Characteristics**				
**Academic Hospital**	64,920 (98.1%)	43,198 (98.3%)	18,111 (99.9%)	3,611 (87.9%)
**Community Hospital**	1,246 (1.9%)	728 (1.7%)	21 (0.1%)	497 (12.1%)
**Outcome Characteristics**				
**CSA-AKI Stage**				
No CSA-AKI	49,264 (74.5%)	33,019 (75.2%)	12,901 (71.2%)	3,344 (81.4%)
CSA-AKI-1	11,759 (17.8%)	7,771 (17.7%)	3,449 (19.0%)	539 (13.1%)
CSA-AKI-2	3,457 (5.2%)	2,161 (4.9%)	1,142 (6.3%)	154 (3.7%)
CSA-AKI-3	1,686 (2.5%)	975 (2.2%)	640 (3.5%)	71 (1.7%)

** Non-mutually exclusive*

Statistics presented as mean (SD) for numeric variables; N(%) for categorical variables. AIDS/HIV = acquired immunodeficiency syndrome / human immunodeficiency virus; AKI = acute kidney injury; ASA = American Society of Anesthesiologists; CPB = cardiopulmonary bypass; CSA-AKI = cardiac surgery-associated acute kidney injury; ETT = endotracheal tube; LMA = laryngeal mask airway

Supplementary Table 3. Description of variables and predictors in the study models

Variable	Description	Variable Type	Category	Valid Range	Phase	Summary statistics	Number of predictors
age	Age (years)	Fixed	Patient demographics		-	-	1
gender	Sex	Fixed	Patient demographics		-	-	1
race	Race	Fixed	Patient demographics		-	-	1
height	Height (cm)	Fixed	Patient demographics		-	-	1
weight	Weight (kg)	Fixed	Patient demographics		-	-	1
bmi	BMI (kg/m2)	Fixed	Patient demographics		-	-	1
smoking_classification	Smoking Classification	Fixed	Patient demographics		-	-	1
asa_class	ASA class	Fixed	Case characteristics		-	-	1
university_affiliated	University affiliation	Fixed	Case characteristics		-	-	1
weekend	Case performed on a weekend	Fixed	Case characteristics		-	-	1
holiday	Case performend on a holiday	Fixed	Case characteristics		-	-	1
emergent	Emergent case ((ASA "E" status)	Fixed	Case characteristics		-	-	1
resident_present	Resident present	Fixed	Case characteristics		-	-	1
crna_present	CRNA present	Fixed	Case characteristics		-	-	1
procedure_type_aortic	Non-hypothermia circulatory arrest aortic	Fixed	Case characteristics		-	-	1
procedure_type_circ_arrest	Circulatory arrest	Fixed	Case characteristics		-	-	1
procedure_type_heart_transplant	Heart transplant	Fixed	Case characteristics		-	-	1
procedure_type_pte	Pulmonary thromboendarterectomy	Fixed	Case characteristics		-	-	1
procedure_type_myectomy	Myectomy	Fixed	Case characteristics		-	-	1
procedure_type_vad	Ventricular assist device (VAD) pre-existing or implanted	Fixed	Case characteristics		-	-	1
procedure_type_iabp	Intra-aortic balloon pumps (IABP) pre-existing or	Fixed	Case characteristics		-	-	1

	placed						
procedure_type_other_mech_support	Other mechanical support device pre-existing or placed	Fixed	Case characteristics		-	-	1
procedure_type_cab_only	Coronary artery bypass (CAB) only	Fixed	Case characteristics		-	-	1
procedure_type_valve_only	Valve replacement only	Fixed	Case characteristics		-	-	1
procedure_type_valve_cab_only	CAB and valve replacement	Fixed	Case characteristics		-	-	1
baseline_bp_map	Baseline BP MAP	Fixed	Pre-operative lab/phys results		-	-	1
preop_platelets	Pre-operative platelets	Fixed	Pre-operative lab/phys results		-	-	1
preop_wbc	Pre-operative WBC	Fixed	Pre-operative lab/phys results		-	-	1
preop_sodium	Pre-operative sodium	Fixed	Pre-operative lab/phys results	[90, 190]	-	-	1
preop_potassium	Pre-operative potassium	Fixed	Pre-operative lab/phys results	[0, 50]	-	-	1
preop_glucose	Pre-operative glucose	Fixed	Pre-operative lab/phys results	[0, 600]	-	-	1
preop_hemoglobin_combined	Pre-operative hemoglobin or hematocrit/3	Fixed	Pre-operative lab/phys results	[0, 30]	-	-	1
preop_hco3_or_co2_serum	Pre-operative bicarbonate or serum CO2	Fixed	Pre-operative lab/phys results	[0, 55]	-	-	1
bl110_count	Anticoagulants	Fixed	Pre-operative home medications		-	-	1
bl117_count	Platelet aggregation inhibitors	Fixed	Pre-operative home medications		-	-	1
cv050_count	Digitalis glycosides	Fixed	Pre-operative home medications		-	-	1
cv100_count	Beta blockers	Fixed	Pre-operative home medications		-	-	1
cv150_count	Alpha blockers	Fixed	Pre-operative home medications		-	-	1
cv200_count	Calcium channel blockers	Fixed	Pre-operative home medications		-	-	1
cv250_count	Anti-anginals	Fixed	Pre-operative home medications		-	-	1
cv300_count	Anti-arrhythmics	Fixed	Pre-operative home medications		-	-	1
cv350_count	Anti-lipemics	Fixed	Pre-operative home medications		-	-	1
cv701_count	Thiazide diuretics	Fixed	Pre-operative home medications		-	-	1

cv702_count	Loop diuretics	Fixed	Pre-operative home medications	-	-	1
cv704_count	Potassium sparing diuretics	Fixed	Pre-operative home medications	-	-	1
cv800_count	Angiotensin-converting enzyme (ACE) inhibitors	Fixed	Pre-operative home medications	-	-	1
cv805_count	Angiotensin II (ATII) inhibitors	Fixed	Pre-operative home medications	-	-	1
hs502_count	Oral hypoglycemic agents	Fixed	Pre-operative home medications	-	-	1
elixhauser_aids_hiv	Elixhauser Comorbidity - AIDS/HIV	Fixed	Comorbidities	-	-	1
elixhauser_alcohol_abuse	Elixhauser Comorbidity - Alcohol abuse	Fixed	Comorbidities	-	-	1
elixhauser_blood_loss_anemia	Elixhauser Comorbidity - Blood loss anemia	Fixed	Comorbidities	-	-	1
elixhauser_cardiac_arrhythmia	Elixhauser Comorbidity - Cardiac arrhythmia	Fixed	Comorbidities	-	-	1
elixhauser_chronic_pulmonary_disease	Elixhauser Comorbidity - Chronic pulmonary disease	Fixed	Comorbidities	-	-	1
elixhauser_coagulopathy	Elixhauser Comorbidity - Coagulopathy	Fixed	Comorbidities	-	-	1
elixhauser_congestive_heart_failure	Elixhauser Comorbidity - Congestive heart failure	Fixed	Comorbidities	-	-	1
elixhauser_deficiency_anemia	Elixhauser Comorbidity - Deficiency anemia	Fixed	Comorbidities	-	-	1
elixhauser_depression	Elixhauser Comorbidity - Depression	Fixed	Comorbidities	-	-	1
elixhauser_diabetes_with_complications	Elixhauser Comorbidity - Diabetes with complications	Fixed	Comorbidities	-	-	1
elixhauser_diabetes_without_complications	Elixhauser Comorbidity - Diabetes without complications	Fixed	Comorbidities	-	-	1
elixhauser_drug_abuse	Elixhauser Comorbidity - Drug abuse	Fixed	Comorbidities	-	-	1
elixhauser_fluid_and_electrolyte_disorders	Elixhauser Comorbidity - Fluid and electrolyte disorders	Fixed	Comorbidities	-	-	1
elixhauser_hypertension_with_complications	Elixhauser Comorbidity - Hypertension with complications	Fixed	Comorbidities	-	-	1
elixhauser_hypertension_without_complications	Elixhauser Comorbidity - Hypertension without complications	Fixed	Comorbidities	-	-	1
elixhauser_hypothyroidism	Elixhauser Comorbidity - Hypothyroidism	Fixed	Comorbidities	-	-	1

elixhauser_liver_disease	Elixhauser Comorbidity - Liver disease	Fixed	Comorbidities	-	-	1
elixhauser_lymphoma	Elixhuaser Comorbidity - Lymphoma	Fixed	Comorbidities	-	-	1
elixhauser_metastatic_cancer	Elixhauser Comorbidity - Metastatic cancer	Fixed	Comorbidities	-	-	1
elixhauser_obesity	Elixhauser Comorbidity - Obesity	Fixed	Comorbidities	-	-	1
elixhauser_other_neurological_disorders	Elixhauser Comorbidity - Other neurological disorders	Fixed	Comorbidities	-	-	1
elixhauser_paralysis	Elixhauser Comorbidity - Paralysis	Fixed	Comorbidities	-	-	1
elixhauser_peptic_ulcer_disease_excluding_bleeding	Elixhauser Comorbidity - Peptic ulcer disease excluding bleeding	Fixed	Comorbidities	-	-	1
elixhauser_peripheral_vascular_disorders	Elixhauser Comorbidity - Peripheral vascular disorders	Fixed	Comorbidities	-	-	1
elixhauser_psychoses	Elixhauser Comorbidity - Psychoses	Fixed	Comorbidities	-	-	1
elixhauser_pulmonary_circulation_disorders	Elixhauser Comorbidity - Pulmonary circulation disorders	Fixed	Comorbidities	-	-	1
elixhauser_rheumatoid_arthritis_collagen	Elixhauser Comorbidity - Rheumatoid arthritis collagen	Fixed	Comorbidities	-	-	1
elixhauser_solid_tumor_without_metastasis	Elixhauser Comorbidity - Solid tumor without metastasis	Fixed	Comorbidities	-	-	1
elixhauser_valvular_disease	Elixhauser Comorbidity - Valvular disease	Fixed	Comorbidities	-	-	1
elixhauser_weight_loss	Elixhauser Comorbidity - Weight loss	Fixed	Comorbidities	-	-	1
preop_creatinine_baseline	Pre-operative baseline sCr (lowest within 60 days prior to surgery)	Fixed	Baseline kidney function	-	-	1
preop_creatinine_most_recent	Pre-operative most recent sCr (closest to surgery, within 60 days prior to surgery)	Fixed	Baseline kidney function	-	-	1
ratio_creatinine_most_recent_to_baseline	Ratio of most recent sCr to baseline sCr	Fixed	Baseline kidney function	-	-	1
diff_creatinine_baseline_to_most_recent	Increase from baseline sCr to most recent sCr	Fixed	Baseline kidney function	-	-	1
baseline_aki_stage	Baseline AKI stage (no AKI, AKI-1, AKI-2, AKI-3)	Fixed	Baseline kidney function	-	-	1
first_postop_creatinine_within_24h	First post-operative sCr within 24h	Fixed	First post-operative sCr	-	-	1

bp_sys	Intra-operative systolic BP	Time-varying	Intra-operative BP	[0, 400]	pre-CPB	first, last, length, min, mean, median, max, slope	8
bp_sys	Intra-operative systolic BP	Time-varying	Intra-operative BP	[0, 400]	intra-CPB	first, last, length, min, mean, median, max, slope	8
bp_sys	Intra-operative systolic BP	Time-varying	Intra-operative BP	[0, 400]	post-CPB	first, last, length, min, mean, median, max, slope	8
bp_dias	Intra-operative diastolic BP	Time-varying	Intra-operative BP	[0, 300]	pre-CPB	first, last, length, min, mean, median, max, slope	8
bp_dias	Intra-operative diastolic BP	Time-varying	Intra-operative BP	[0, 300]	intra-CPB	first, last, length, min, mean, median, max, slope	8
bp_dias	Intra-operative diastolic BP	Time-varying	Intra-operative BP	[0, 300]	post-CPB	first, last, length, min, mean, median, max, slope	8
bp_map	Intra-operative MAP	Time-varying	Intra-operative BP	[0, 200]	pre-CPB	first, last, length, min, mean, median, max, slope	8
bp_map	Intra-operative MAP	Time-varying	Intra-operative BP	[0, 200]	intra-CPB	first, last, length, min, mean, median, max, slope	8
bp_map	Intra-operative MAP	Time-varying	Intra-operative BP	[0, 200]	post-CPB	first, last, length, min, mean, median, max, slope	8
cvp	Intra-operative CVP	Time-varying	Intra-operative CVP	[-10, 40]	pre-CPB	first, last, length, min, mean, median, max, slope	8
cvp	Intra-operative CVP	Time-varying	Intra-operative CVP	[-10, 40]	intra-CPB	first, last, length, min, mean, median, max, slope	8

cvp	Intra-operative CVP	Time-varying	Intra-operative CVP	[-10, 40]	post-CPB	first, last, length, min, mean, median, max, slope	8
Bicarbonate	Intra-operative bicarbonate	Time-varying	Intra-operative lab	[0, 55]	pre-CPB	first, last, length, min, mean, median, max, slope	8
Bicarbonate	Intra-operative bicarbonate	Time-varying	Intra-operative lab	[0, 55]	intra-CPB	first, last, length, min, mean, median, max, slope	8
Bicarbonate	Intra-operative bicarbonate	Time-varying	Intra-operative lab	[0, 55]	post-CPB	first, last, length, min, mean, median, max, slope	8
Glucose	Intra-operative glucose	Time-varying	Intra-operative lab	[0, 600]	pre-CPB	first, last, length, min, mean, median, max, slope	8
Glucose	Intra-operative glucose	Time-varying	Intra-operative lab	[0, 600]	intra-CPB	first, last, length, min, mean, median, max, slope	8
Glucose	Intra-operative glucose	Time-varying	Intra-operative lab	[0, 600]	post-CPB	first, last, length, min, mean, median, max, slope	8
Hemoglobin	Intra-operative hemoglobin	Time-varying	Intra-operative lab	[0, 30]	pre-CPB	first, last, length, min, mean, median, max, slope	8
Hemoglobin	Intra-operative hemoglobin	Time-varying	Intra-operative lab	[0, 30]	intra-CPB	first, last, length, min, mean, median, max, slope	8
Hemoglobin	Intra-operative hemoglobin	Time-varying	Intra-operative lab	[0, 30]	post-CPB	first, last, length, min, mean, median, max, slope	8
pCO2	Intra-operative pCO2	Time-varying	Intra-operative lab	[0, 200]	pre-CPB	first, last, length, min, mean, median, max, slope	8

pCO2	Intra-operative pCO2	Time-varying	Intra-operative lab	[0, 200]	intra-CPB	first, last, length, min, mean, median, max, slope	8
pCO2	Intra-operative pCO2	Time-varying	Intra-operative lab	[0, 200]	post-CPB	first, last, length, min, mean, median, max, slope	8
pH	Intra-operative pH	Time-varying	Intra-operative lab	[6.7, 8]	pre-CPB	first, last, length, min, mean, median, max, slope	8
pH	Intra-operative pH	Time-varying	Intra-operative lab	[6.7, 8]	intra-CPB	first, last, length, min, mean, median, max, slope	8
pH	Intra-operative pH	Time-varying	Intra-operative lab	[6.7, 8]	post-CPB	first, last, length, min, mean, median, max, slope	8
Potassium	Intra-operative potassium	Time-varying	Intra-operative lab	[0, 50]	pre-CPB	first, last, length, min, mean, median, max, slope	8
Potassium	Intra-operative potassium	Time-varying	Intra-operative lab	[0, 50]	intra-CPB	first, last, length, min, mean, median, max, slope	8
Potassium	Intra-operative potassium	Time-varying	Intra-operative lab	[0, 50]	post-CPB	first, last, length, min, mean, median, max, slope	8
Sodium	Intra-operative sodium	Time-varying	Intra-operative lab	[90, 190]	pre-CPB	first, last, length, min, mean, median, max, slope	8
Sodium	Intra-operative sodium	Time-varying	Intra-operative lab	[90, 190]	intra-CPB	first, last, length, min, mean, median, max, slope	8
Sodium	Intra-operative sodium	Time-varying	Intra-operative lab	[90, 190]	post-CPB	first, last, length, min, mean, median, max, slope	8

Albuterol	Intra-operative administration of albuterol	Time-varying	Intra-operative medication	pre-CPB	length	1
Albuterol	Intra-operative administration of albuterol	Time-varying	Intra-operative medication	intra-CPB	length	1
Albuterol	Intra-operative administration of albuterol	Time-varying	Intra-operative medication	post-CPB	length	1
Angiotension II	Intra-operative administration of angiotension II	Time-varying	Intra-operative medication	pre-CPB	length	1
Angiotension II	Intra-operative administration of angiotension II	Time-varying	Intra-operative medication	intra-CPB	length	1
Angiotension II	Intra-operative administration of angiotension II	Time-varying	Intra-operative medication	post-CPB	length	1
Dobutamine	Intra-operative administration of dobutamine	Time-varying	Intra-operative medication	pre-CPB	length	1
Dobutamine	Intra-operative administration of dobutamine	Time-varying	Intra-operative medication	intra-CPB	length	1
Dobutamine	Intra-operative administration of dobutamine	Time-varying	Intra-operative medication	post-CPB	length	1
Dopamine	Intra-operative administration of dopamine	Time-varying	Intra-operative medication	pre-CPB	length	1
Dopamine	Intra-operative administration of dopamine	Time-varying	Intra-operative medication	intra-CPB	length	1
Dopamine	Intra-operative administration of dopamine	Time-varying	Intra-operative medication	post-CPB	length	1
Ephedrine	Intra-operative administration of ephedrine	Time-varying	Intra-operative medication	pre-CPB	length	1
Ephedrine	Intra-operative administration of ephedrine	Time-varying	Intra-operative medication	intra-CPB	length	1
Ephedrine	Intra-operative administration of ephedrine	Time-varying	Intra-operative medication	post-CPB	length	1
Epinephrine	Intra-operative administration of epinephrine	Time-varying	Intra-operative medication	pre-CPB	length	1
Epinephrine	Intra-operative administration of epinephrine	Time-varying	Intra-operative medication	intra-CPB	length	1
Epinephrine	Intra-operative administration of epinephrine	Time-varying	Intra-operative medication	post-CPB	length	1

Milrinone	Intra-operative administration of milrinone	Time-varying	Intra-operative medication		pre-CPB	length	1
Milrinone	Intra-operative administration of milrinone	Time-varying	Intra-operative medication		intra-CPB	length	1
Milrinone	Intra-operative administration of milrinone	Time-varying	Intra-operative medication		post-CPB	length	1
Norepinephrine	Intra-operative administration of norepinephrine	Time-varying	Intra-operative medication		pre-CPB	length	1
Norepinephrine	Intra-operative administration of norepinephrine	Time-varying	Intra-operative medication		intra-CPB	length	1
Norepinephrine	Intra-operative administration of norepinephrine	Time-varying	Intra-operative medication		post-CPB	length	1
Phenylephrine	Intra-operative administration of phenylephrine	Time-varying	Intra-operative medication		pre-CPB	length	1
Phenylephrine	Intra-operative administration of phenylephrine	Time-varying	Intra-operative medication		intra-CPB	length	1
Phenylephrine	Intra-operative administration of phenylephrine	Time-varying	Intra-operative medication		post-CPB	length	1
Vasopressin	Intra-operative administration of vasopressin	Time-varying	Intra-operative medication		pre-CPB	length	1
Vasopressin	Intra-operative administration of vasopressin	Time-varying	Intra-operative medication		intra-CPB	length	1
Vasopressin	Intra-operative administration of vasopressin	Time-varying	Intra-operative medication		post-CPB	length	1
HR	Intra-operative heart rate	Time-varying	Intra-operative physiology	[30, 180]	pre-CPB	first, last, length, min, mean, median, max, slope	8
HR	Intra-operative heart rate	Time-varying	Intra-operative physiology	[30, 180]	intra-CPB	first, last, length, min, mean, median, max, slope	8
HR	Intra-operative heart rate	Time-varying	Intra-operative physiology	[30, 180]	post-CPB	first, last, length, min, mean, median, max, slope	8

SpO2	Intra-operative SpO2	Time-varying	Intra-operative physiology	[60, 100]	pre-CPB	first, last, length, min, mean, median, max, slope	8
SpO2	Intra-operative SpO2	Time-varying	Intra-operative physiology	[60, 100]	intra-CPB	first, last, length, min, mean, median, max, slope	8
SpO2	Intra-operative SpO2	Time-varying	Intra-operative physiology	[60, 100]	post-CPB	first, last, length, min, mean, median, max, slope	8
**Total**							426
